# Supplementary material for: Potential of the Oxidized Form of the Oleuropein Aglycon to Monitor the Oil Quality Evolution of Commercial Extra-Virgin Olive Oils
Source: Foods. 2023 Aug 4;12(15):2959. doi: 10.3390/foods12152959 (PMC10418756; doi:10.3390/foods12152959)
Supplement: Supplementary file 1 [file foods-12-02959-s001.zip › Figure S2.pdf]

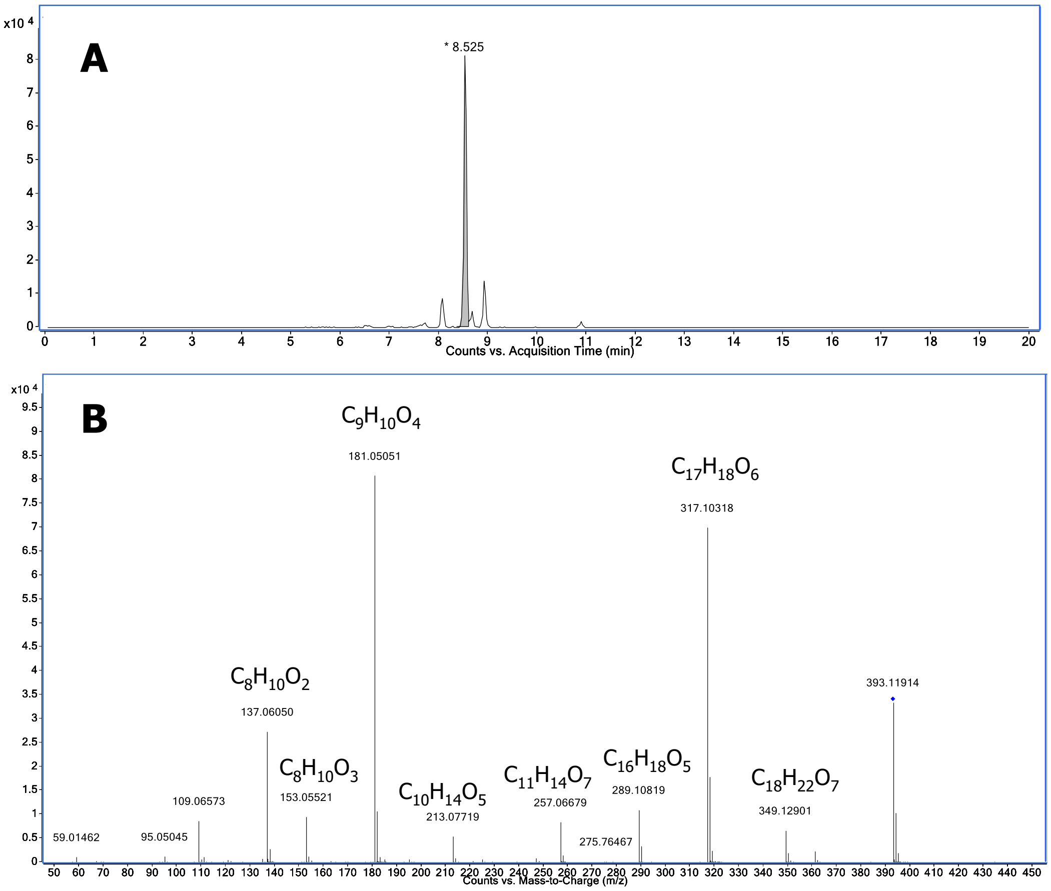

Figure S2: LC-MS-MS chromatogram (A) and LC-MS-MS spectrum (B) of one of the 20 VOOs (specifically S2) after 12 months with light exposure.
