# Supplementary material for: Potential of the Oxidized Form of the Oleuropein Aglycon to Monitor the Oil Quality Evolution of Commercial Extra-Virgin Olive Oils
Source: Foods. 2023 Aug 4;12(15):2959. doi: 10.3390/foods12152959 (PMC10418756; doi:10.3390/foods12152959)
Supplement: Supplementary file 1 [file foods-12-02959-s001.zip › Figure S3.pdf]

Score plot: t[1]/t[2]

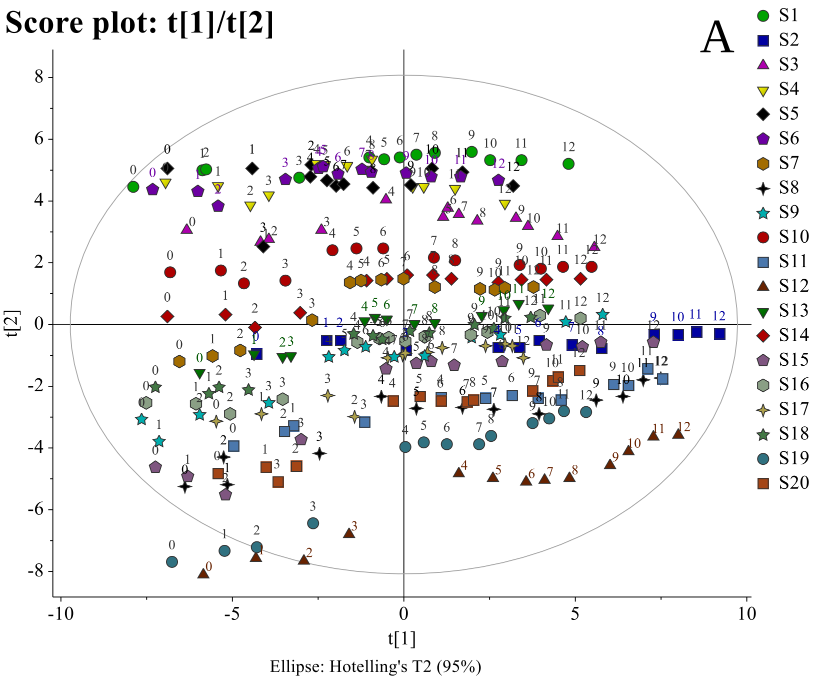

Loading plot: p[1]/p[2]

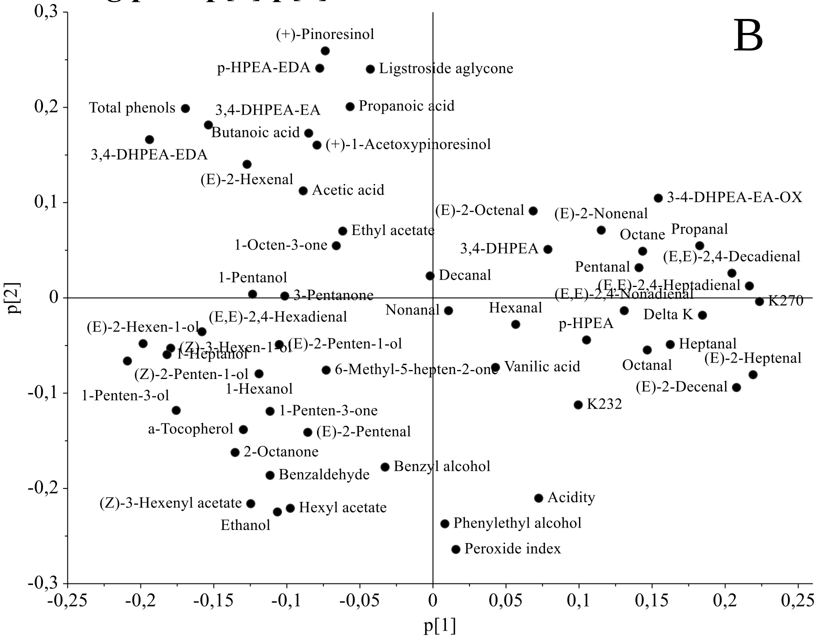

Figure S3: Score plot (t_1/t_2) (**A**) and loading plot (p_1/p_2) (**B**) of the first two principal components of the PCA model built using all the analytical evaluations carried out on the 20 VOOs exposed to light for 12 months. The PCA model explains 70% of the total variance with six significant principal components (27%, 19%, 9%, 7%, 5%, and 3%, respectively).
