# Supplementary material for: Potential of the Oxidized Form of the Oleuropein Aglycon to Monitor the Oil Quality Evolution of Commercial Extra-Virgin Olive Oils
Source: Foods. 2023 Aug 4;12(15):2959. doi: 10.3390/foods12152959 (PMC10418756; doi:10.3390/foods12152959)
Supplement: Supplementary file 1 [file foods-12-02959-s001.zip › Figure S4.pdf]

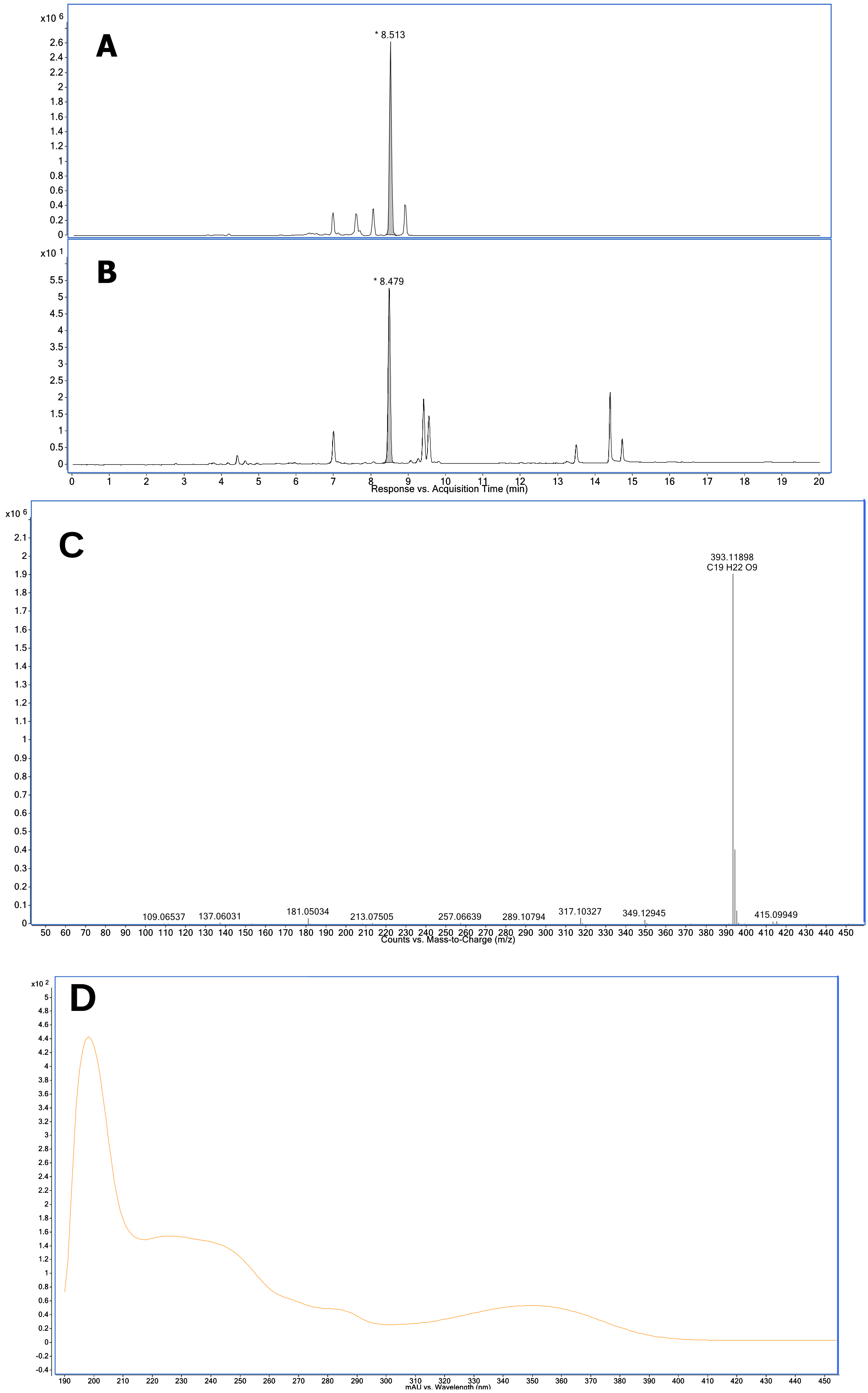

Figure S4: Chromatograms of one of the 20 VOOs (specifically S2) after 12 months of light exposure (A, B); LC-MS extracted ion chromatogram in negative mode (393.11911 m/z) showing the acidic form of oleuropein aglycone (C); DAD-UV chromatogram absorption at a wavelength of 347 nm and at a retention time of at 8.479 min (D).
