# Supplementary material for: Potential of the Oxidized Form of the Oleuropein Aglycon to Monitor the Oil Quality Evolution of Commercial Extra-Virgin Olive Oils
Source: Foods. 2023 Aug 4;12(15):2959. doi: 10.3390/foods12152959 (PMC10418756; doi:10.3390/foods12152959)
Supplement: Supplementary file 1 [file foods-12-02959-s001.zip › Table S1.pdf]

Table S1: Mixing proportions of the 6 "mother" samples A, C, D, E, F, and G selected for the preparation of the 20 experimental VOOs S1-S20

S1	100 % A
S2	100 % C
S3	75 % A + 25 % C
S4	25 % A + 75 % D
S5	50 % A + 50 % D
S6	75 % A + 25 % D
S7	25 % A + 75 % E
S8	25 % A + 75 % G
S9	50 % A + 50 % G
S10	25 % C + 75 % D
S11	75 % C + 25 % F
S12	25 % C + 75 % G
S13	25 % D + 75 % F
S14	50 % D + 50 % F
S15	25 % D + 75 % G
S16	50 % D + 50 % G
S17	25 % E + 75 % F
S18	75 % E + 25 % F
S19	25 % F + 75 % G
S20	75 % F + 25 % G
