# Supplementary material for: Potential of the Oxidized Form of the Oleuropein Aglycon to Monitor the Oil Quality Evolution of Commercial Extra-Virgin Olive Oils
Source: Foods. 2023 Aug 4;12(15):2959. doi: 10.3390/foods12152959 (PMC10418756; doi:10.3390/foods12152959)
Supplement: Supplementary file 1 [file foods-12-02959-s001.zip › Table S10.pdf]

Table S10: Evolution of the oxidized form of oleuropein aglycon (3,4-DHPEA-EA-OX) over 12 month storage with light exposure in VOOlmp and VOOmhp samples*

Time (months)		0	1	2	3	4	5	6	7	8	9	10	11	12
VOOlmp	S13	n.d.	6.69 (0.02) a	6.7 (0.01) a	6.68 (0.03) a	7.5 (0.3) b	9.3 (0.2) c	11.1 (0.1) d	13.7 (0.1) e	13.73 (0.03) e	16.6 (0.2) f	24.7 (0.1) g	25.2 (0.3) gh	25.5 (0.1) h
	S7	1.50 (0.06) a	1.7 (0.4) a	9.0 (0.5) b	9.7 (0.4) bc	10.7 (0.1) c	10.94 (0.03) cd	12.0 (0.1) d	14.6 (0.1) e	16.7 (0.5) f	20.0 (0.5) g	21.4 (0.4) h	24.4 (0.4) i	24.7 (0.1) i
	S2	0.371 (0.001) a	8.865 (0.009) b	15.1 (0.01) c	17.14 (0.01) d	18.4 (0.1) e	18.27 (0.05) e	23.9 (0.2) f	26.9 (0.3) g	30.0 (0.3) h	36.9 (0.2) i	40.0 (0.3) l	44.5 (0.1) m	44.3 (0.1) m
	S8	1.998 (0.006) a	3.83 (0.01) b	6.8 (0.02) c	6.79 (0.02) c	7.3 (0.2) d	8.12 (0.01) e	10.20 (0.04) f	12.8 (0.1) g	18.1 (0.2) h	20.6 (0.3) i	23.8 (0.1) l	24.1 (0.1) l	23.92 (0.04) l
	S18	n.d.	10.31 (0.03) a	11.5 (0.06) b	11.50 (0.04) b	11.54 (0.03) b	12.12 (0.05) c	16.83 (0.02) d	16.6 (0.3) d	15.6 (0.1) e	15.3 (0.2) e	15.3 (0.1) e	10.4 (0.3) a	8.9 (0.2) h
	S11	1.31 (0.02) a	3.48 (0.06) a	3.3 (0.06) b	5.35 (0.08) c	6.58 (0.02) d	13.07 (0.09) e	18.1 (0.2) f	21.4 (0.5) g	23.48 (0.03) h	28.4 (0.1) i	29.93 (0.04) l	28.1 (0.2) i	28.2 (0.2) i
	S17	n.d.	7.30 (0.02) a	8.8 (0.03) b	8.78 (0.02) b	8.7 (0.2) b	8.76 (0.04) b	8.6 (0.2) bc	8.38 (0.02) bcd	8.4 (0.1) cd	8.1 (0.1) de	7.9 (0.1) e	7.2 (0.1) a	6.48 (0.01) f
	S19	1.201 (0.004) a	4.23 (0.01) b	6.5 (0.02) c	6.54 (0.02) c	6.9 (0.2) c	7.05 (0.09) cd	7.8 (0.2) d	9.0 (0.1) e	3.8 (0.6) bf	3.4 (0.3) f	3.1 (0.1) f	2.1 (0.2) gh	1.4 (0.1) ah
	S20	0.519 (0.002) a	6.21 (0.02) b	8.6 (0.02) ce	8.59 (0.02) ce	8.3 (0.2) cf	9.56 (0.01) d	8.9 (0.3) e	8.5 (0.2) cef	8.0 (0.1) f	7.4 (0.2) g	7.03 (0.07) gh	6.7 (0.1) bh	6.4 (0.2) b
	S12	0.972 (0.003) a	6.79 (0.02) b	4.8 (0.02) c	4.80 (0.01) c	2.5 (0.1) def	2.54 (0.04) def	2.41 (0.01) def	2.7 (0.1) d	3.0 (0.1) d	3.0 (0.1) d	2.1 (0.2) def	2.1 (0.2) ef	1.6 (0.8) af
VOOmhp	S1	0.94 (0.03) a	5.8 (0.1) b	8.3 (0.1) c	12.28 (0.03) d	16.2 (0.4) e	18.6 (0.1) f	18.74 (0.01) f	19.7 (0.3) f	24.4 (0.5) g	30.8 (0.1) h	36.1 (0.1) i	45.2 (0.1) l	58.7 (1.2) m
	S5	n.d.	5.56 (0.02) a	5.75 (0.02) a	5.70 (0.03) a	6.4 (0.3) a	8.5 (0.2) b	9.39 (0.03) bc	9.9 (0.1) c	12.3 (0.9) d	12.82 (0.03) d	34.18 (0.02) e	44.35 (0.05) f	55.7 (0.1) g
	S4	n.d.	1.48 (0.02) a	1.74 (0.02) a	2.41 (0.02) a	4.5 (0.1) b	4.8 (0.1) b	4.7 (0.2) b	8.6 (0.1) c	8.8 (0.4) c	10.53 (0.08) d	14.58 (0.08) e	22.6 (0.1) f	35.7 (0.7) g
	S6	n.d.	2.93 (0.02) a	10.17 (0.02) b	14.20 (0.02) c	15.4 (0.1) cd	15.8 (0.4) d	16.1 (0.3) d	18.79 (0.05) f	19.9 (1.2) f	24.1 (0.1) g	36.08 (0.03) h	47.3 (0.2) i	58.8 (0.2) l
	S10	0.65 (0.05) a	2.0 (0.2) b	2.8 (0.2) bc	3.8 (0.3) c	9.0 (0.3) d	12.9 (0.2) e	15.03 (0.03) f	17.5 (0.2) g	25.7 (0.8) h	28.3 (0.1) i	36.4 (0.1) l	40.50 (0.06) m	40.1 (0.3) n
	S3	0.809 (0.003)	4.25 (0.01) b	4.25 (0.01) b	6.30 (0.02) c	9.3 (0.2) d	13.6 (0.1) e	14.75 (0.04) f	16.85 (0.06) g	20.4 (0.3) h	28.95 (0.08) i	30.04 (0.02) l	45.29 (0.08) m	53.30 (0.08) n
	S14	0.634 (0.002) a	16.77 (0.02) b	21.79 (0.02) c	22.02 (0.02) c	22.98 (0.04) c	24.75 (0.01) d	25.47 (0.03) d	28.4 (0.3) e	30.2 (1.1) f	31.72 (0.08) g	38.83 (0.05) h	50.3 (0.1) i	55.7 (0.4) l
	S16	1.074 (0.003) a	7.16 (0.02) b	10.46 (0.02) c	12.50 (0.02) d	13.7 (0.6) e	13.7 (0.2) e	16.60 (0.02) f	17.9 (0.4) g	24.3 (0.1) h	25.4 (0.5) i	26.7 (0.2) l	41.91 (0.07) m	48.6 (0.2) n
	S9	0.781 (0.002) a	1.04 (0.01) a	5.02 (0.02) b	8.02 (0.02) c	8.6 (0.2) c	10.9 (0.1) d	11.3 (0.4) d	13.0 (0.3) e	13.4 (0.1) e	14.6 (0.5) f	25.0 (0.2) g	26.2 (0.1) h	30.1 (0.2) i
	S15	1.699 (0.005) a	8.13 (0.03) b	15.93 (0.02) c	15.90 (0.01) c	15.1 (0.3) d	16.14 (0.01) c	17.0 (0.1) d	17.4 (0.3) d	17.1 (0.2) d	18.2 (0.1) e	21.5 (0.1) f	35.0 (0.1) g	40.6 (0.5) h

*The results are the means of two independent determinations ± standard deviation. Different letters in each row indicate statistically different values at $p < 0.05$. Legend: VOOlmp: Virgin olive oil with low-medium poly-phenol content; VOOmhp: Virgin olive oil with medium-high polyphenol content. N.d.: Not detected.
