# Supplementary material for: Potential of the Oxidized Form of the Oleuropein Aglycon to Monitor the Oil Quality Evolution of Commercial Extra-Virgin Olive Oils
Source: Foods. 2023 Aug 4;12(15):2959. doi: 10.3390/foods12152959 (PMC10418756; doi:10.3390/foods12152959)
Supplement: Supplementary file 1 [file foods-12-02959-s001.zip › Table S11.pdf]

Table S11: Evolution of the oleuropein aglycon (3,4-DHPEA-EA) over 12 month storage with light exposure in VOOlmp and VOOmhp samples*

Time (months)		0	1	2	3	4	5	6	7	8	9	10	11	12
VOOlmp	S13	137.7 (0.3) a	128.6 (2.0) ab	123.3 (1.2) bc	121.2 (0.5) bc	115.5 (1.2) cd	107.4 (5.0) de	98.8 (4.3) e	65.1 (0.6) f	75.4 (5.4) f	52.1 (1.6) g	36.2 (0.1) h	n.d.	n.d.
	S7	104.0 (0.7) a	102.7 (3.7) a	88.3 (2.0) b	81.6 (4.8) bd	77.0 (0.5) d	75.0 (0.9) de	66.7 (1.5) e	53.8 (3.2) f	34.7 (0.7) g	12.2 (2.9) h	8.5 (1.3) h	n.d.	n.d.
	S2	206.5 (2.4) a	170.5 (1.4) b	136.7 (2.6) c	105.0 (9.2) d	94.6 (0.6) de	91.1 (0.1) e	86.7 (1.2) ef	74.3 (0.9) f	55.7 (2.8) g	33.5 (2.9) h	14.9 (0.2) i	n.d.	n.d.
	S8	122.5 (0.3) a	108.6 (4.0) b	93.2 (1.4) c	90.4 (0.5) c	85.7 (1.5) cd	76.9 (1.5) d	65.6 (3.8) e	59.4 (1.8) e	38.9 (3.6) f	14.7 (2.0) g	6.3 (0.2) g	n.d.	n.d.
	S18	32.5 (0.1) a	30.2 (1.8) ab	26.8 (0.9) bc	24.1 (0.3) c	23.6 (0.1) c	n.d.	n.d.	n.d.	n.d.	n.d.	n.d.	n.d.	n.d.
	S11	122.01 (0.01) a	117.2 (6.5) a	100.9 (3.0) b	93.3 (2.4) bc	89.8 (0.7) c	68.1 (1.5) d	47.5 (2.5) e	30.4 (0.7) f	18.4 (1.3) g	n.d.	n.d.	n.d.	n.d.
	S17	53.5 (0.5) a	41.4 (0.7) b	37.5 (0.1) c	23.8 (1.8) d	22.4 (0.4) d	n.d.	n.d.	n.d.	n.d.	n.d.	n.d.	n.d.	n.d.
	S19	62.3 (0.6) a	58.2 (1.8) a	25.9 (0.2) b	23.4 (2.4) bc	21.4 (0.1) bcd	19.4 (0.6) cd	17.3 (0.4) d	n.d.	n.d.	n.d.	n.d.	n.d.	n.d.
	S20	22.46 (0.01) a	17.8 (0.9) b	13.9 (0.2) c	8.9 (0.4) d	n.d.	n.d.	n.d.	n.d.	n.d.	n.d.	n.d.	n.d.	n.d.
	S12	67.1 (1.4) a	61.7 (0.2) a	55.2 (1.2) b	53.3 (0.7) b	46.2 (2) c	34.9 (2.7) d	25.4 (1.3) e	n.d.	n.d.	n.d.	n.d.	n.d.	n.d.
VOOmhp	S1	276.9 (19.2) a	258.1 (5.5) ab	244.6 (0.3) bc	226.7 (15.6) c	204.5 (1.3) de	198.3 (0.2) def	196.9 (4.5) ef	181.1 (2.2) f	144.9 (0.2) g	126.2 (1.6) gh	113.0 (0.4) h	86.7 (2.4) i	60.5 (3.3) l
	S5	339.0 (1.1) a	326.3 (9.5) ab	324.2 (9.0) ab	311.6 (1.5) bc	294.2 (1.1) cd	286.3 (3.3) de	275.9 (2.3) def	266.9 (9.9) efg	259.9 (2.2) fg	250.0 (6.5) g	209.3 (1.2) h	158.2 (1.5) i	86.8 (2.4) l
	S4	352.4 (2.4) a	347.9 (5.8) a	332.7 (2.7) bc	326.3 (8.6) cd	316.2 (0.9) de	307.4 (0.9) ef	300.3 (0.9) f	295.4 (4.2) f	277.3 (4.4) g	258.0 (0.9) i	224.3 (4.1) l	174.0 (0.5) m	78.2 (0.6) n
	S6	309.4 (2.1) a	305.5 (4.1) a	278.2 (3.7) b	276.3 (9.7) b	269.1 (1.2) bc	258.5 (1.2) cd	242.3 (1.1) de	232.7 (3.1) ef	224.8 (10.2) fg	211.7 (2) g	178.0 (0.2) h	142.3 (0.5) i	98 (0.5) l
	S10	325.9 (1.7) a	322.7 (4.5) ab	314.3 (0.2) b	295.9 (2.3) c	258.1 (1.5) d	235.4 (0.3) e	218.6 (2.2) g	176.1 (2.2) h	148.6 (2.1) i	96.8 (3.8) l	51.1 (0.4) m	38.2 (0.7) n	7.2 (0.6) o
	S3	252.2 (0.9) a	224.6 (2.1) b	220.4 (3.1) bc	206.8 (1.2) c	179.5 (1.4) d	146.9 (11.0) e	125.4 (1.2) f	101.5 (1.9) h	82.8 (1.4) i	57.9 (1.9) l	48.9 (0.1) l	28.2 (0.8) m	n.d.
	S14	243.8 (1.3) a	213.2 (2.3) b	188.4 (4.7) c	186.7 (1.8) c	181.4 (5.0) cd	178.5 (3.3) cd	173.2 (3.5) d	160.4 (0.1) e	145.1 (0.5) f	131.3 (1.2) g	110.3 (0.1) h	53.6 (2.0) i	29.1 (0.6) l
	S16	244.8 (0.9) a	241.1 (1.4) a	208.2 (3.9) b	203.2 (4.9) b	198.9 (2.9) bc	189.2 (2.8) cd	179.2 (3.5) de	170.9 (1.2) e	158.9 (3) f	136.5 (4.7) g	114.4 (0.3) h	83.2 (0.8) i	48.9 (2.5) l
	S9	174.2 (0.6) a	172.5 (1.3) a	163.8 (0.4) a	130.9 (7.4) b	127.0 (0.7) b	119.8 (1.0) bd	116.7 (0.4) d	102.3 (0.4) e	94.9 (4.1) ef	85.1 (4.9) f	47.5 (0.9) g	25.2 (2.1) h	6.4 (0.3) i
	S15	200.9 (2.2) a	167.4 (3.7) b	134.0 (0.6) c	129.8 (0.1) cd	124.7 (0.2) de	121.4 (2.8) e	110.9 (0.8) f	109.6 (2.3) f	97.8 (1.6) g	93.1 (1.9) g	68.9 (0.2) h	12.7 (1.9) i	n.d.

*The results are the means of two independent determinations ± standard deviation. Different letters in each row indicate statistically different values at $p < 0.05$. Legend: VOOlmp: Virgin olive oil with low-medium poly-phenol content; VOOmhp: Virgin olive oil with medium-high polyphenol content. N.d.: Not detected.
