# Supplementary material for: Potential of the Oxidized Form of the Oleuropein Aglycon to Monitor the Oil Quality Evolution of Commercial Extra-Virgin Olive Oils
Source: Foods. 2023 Aug 4;12(15):2959. doi: 10.3390/foods12152959 (PMC10418756; doi:10.3390/foods12152959)
Supplement: Supplementary file 1 [file foods-12-02959-s001.zip › Table S2.pdf]

Table S2: Values of free acidity, peroxide value, extinction coefficients, 1,2 diacylglycerols, oleic acid, MUFA, PUFA and SFA, of the 20 VOOs at time at time 0*

	Free acidity (%)	Peroxide value (meq O_2/kg)	K_{232}	K_{270}	ΔK	1,2 Diacylglycerols (%)	Oleic acid (%)	MUFA (%)	PUFA (%)	SFA (%)
S1	0.25 (0.01)	6.3 (0.5)	1.87 (0.09)	0.134 (0.003)	-0.0035 (0.0001)	80.7 (4.8)	80.48 (1.87)	81.11 (1.87)	6.19 (0.51)	12.71 (0.03)
S2	0.34 (0.01)	10.7 (0.3)	2.26 (0.08)	0.129 (0.004)	-0.0020 (0.00005)	34.7 (1.6)	54.95 (1.08)	57.61 (1.11)	19.99 (1.39)	22.40 (0.02)
S3	0.28 (0.01)	7.2 (0.1)	2.03 (0.08)	0.132 (0.004)	-0.0030 (0.0001)	94.8 (5.8)	74.84 (0.03)	76.04 (0.05)	9.39 (0.02)	14.57 (0.11)
S4	0.27 (0.01)	4.8 (0.2)	1.76 (0.06)	0.132 (0.003)	-0.0040 (0.0001)	75.0 (4.1)	77.64 (1.32)	78.95 (1.32)	5.78 (0.18)	15.27 (0.50)
S5	0.25 (0.01)	4.3 (0.1)	1.76 (0.07)	0.125 (0.002)	-0.0045 (0.0001)	72.1 (3.3)	79.22 (0.43)	79.96 (0.43)	5.81 (0.03)	14.23 (0.09)
S6	0.25 (0.01)	5.2 (0.3)	1.78 (0.05)	0.106 (0.003)	-0.0035 (0.0002)	78.0 (5.3)	79.76 (0.13)	80.69 (0.13)	6.06 (0.05)	13.26 (0.09)
S7	0.25 (0.01)	6.0 (0.2)	1.81 (0.06)	0.132 (0.005)	-0.0020 (0.0001)	72.5 (4.3)	75.66 (0.20)	77.11 (0.21)	8.13 (0.01)	14.76 (0.02)
S8	0.37 (0.01)	8.8 (0.4)	1.85 (0.07)	0.133 (0.004)	-0.0045 (0.0002)	52.2 (1.9)	78.17 (0.23)	78.95 (0.23)	6.8 (0.02)	14.25 (0.27)
S9	0.34 (0.02)	7.9 (0.1)	1.84 (0.05)	0.133 (0.004)	-0.0045 (0.0002)	62.6 (3.8)	78.77 (0.07)	79.70 (0.07)	6.63 (0.02)	13.67 (0.07)
S10	0.27 (0.01)	6.0 (0.2)	1.82 (0.05)	0.118 (0.005)	-0.0040 (0.0002)	66.9 (3.2)	72.79 (0.01)	74.33 (0.03)	8.71 (0.02)	16.96 (0.03)
S11	0.33 (0.03)	10.6 (0.5)	2.23 (0.10)	0.124 (0.003)	-0.0025 (0.0001)	59.2 (4.1)	58.18 (1.64)	60.70 (1.64)	17.89 (0.69)	21.41 (0.71)
S12	0.42 (0.02)	11.0 (0.3)	2.01 (0.06)	0.125 (0.004)	-0.0050 (0.0002)	54.8 (2.2)	71.59 (0.68)	73.01 (0.68)	10.06 (0.31)	16.93 (0.26)
S13	0.28 (0.01)	7.9 (0.3)	2.02 (0.08)	0.115 (0.003)	-0.0050 (0.0002)	71.2 (4.0)	70.35 (0.03)	72.26 (0.03)	10.63 (0.03)	17.11 (0.04)
S14	0.28 (0.01)	6.8 (0.2)	1.90 (0.08)	0.113 (0.003)	-0.0055 (0.0003)	72.6 (5.1)	72.4 (0.48)	74.36 (0.48)	8.92 (0.13)	16.72 (0.21)
S15	0.44 (0.02)	8.9 (0.3)	1.75 (0.08)	0.125 (0.004)	-0.0050 (0.0002)	56.4 (2.8)	77.2 (0.23)	78.72 (0.23)	6.447 (0.004)	14.83 (0.12)
S16	0.35 (0.01)	7.6 (0.2)	1.70 (0.07)	0.122 (0.005)	-0.0055 (0.0002)	60.7 (3.6)	77.48 (0.05)	78.66 (0.05)	6.21 (0.01)	15.13 (0.11)
S17	0.28 (0.01)	9.3 (0.3)	2.05 (0.09)	0.109 (0.003)	-0.0050 (0.0002)	68.0 (3.3)	68.93 (0.49)	71.08 (0.51)	11.48 (0.14)	17.44 (0.06)
S18	0.22 (0.01)	7.1 (0.1)	1.81 (0.04)	0.094 (0.003)	-0.0030 (0.0001)	76.8 (5.4)	72.44 (0.14)	74.14 (0.15)	9.77 (0.07)	16.09 (0.17)
S19	0.39 (0.02)	10.2 (0.2)	1.90 (0.06)	0.125 (0.004)	-0.0050 (0.0001)	56.1 (2.8)	74.78 (0.10)	76.25 (0.11)	8.30 (0.02)	15.45 (0.09)
S20	0.31 (0.01)	9.4 (0.3)	2.09 (0.09)	0.120 (0.003)	-0.0055 (0.0002)	64.4 (2.6)	69.96 (0.03)	71.90 (0.03)	11.10 (0.02)	17.01 (0.02)

*The results are the means of two independent determinations ± standard deviation. Legend S: sample; MUFA: Monounsaturated Fatty acids; PUFA: Polyunsaturated Fatty Acids; SFA: Saturated Fatty Acids.
