# Supplementary material for: Potential of the Oxidized Form of the Oleuropein Aglycon to Monitor the Oil Quality Evolution of Commercial Extra-Virgin Olive Oils
Source: Foods. 2023 Aug 4;12(15):2959. doi: 10.3390/foods12152959 (PMC10418756; doi:10.3390/foods12152959)
Supplement: Supplementary file 1 [file foods-12-02959-s001.zip › Table S3.pdf]

Table S3: Volatiles' composition (μg/kg) of the head space of the 20 VOOs at time 0*

	S1	S2	S3	S4	S5	S6	S7	S8	S9	S10	S11	S12	S13	S14	S15	S16	S17	S18	S19	S20
**Aldehydes**																				
Propanal	93.9 (0.2)	104.6 (0.8)	73 (2)	43 (1)	51.2 (0.4)	62 (1)	100 (1)	74.4 (0.5)	78 (1)	45 (1)	69 (1)	76 (2)	50 (1)	46.7 (0.3)	63.4 (0.3)	57.3 (0.2)	54.9 (0.2)	54 (2)	79 (9)	58 (2)
Pentanal	36 (6)	35 (3)	44 (1)	50.9 (0.1)	30.1 (0.2)	38 (3)	44.8 (0.4)	38.1 (0.2)	40.6 (0.2)	31 (1)	34 (1)	36 (1)	21 (1)	26 (6)	37 (1)	33 (5)	23 (2)	28 (1)	37 (1)	26.5 (0.1)
(E)-2-Pentenal	38 (1)	48.0 (0.3)	37.2 (0.4)	31 (1)	32 (1)	35.0 (0.1)	35.3 (0.2)	44 (1)	43.4 (0.2)	38.7 (0.6)	50.3 (0.4)	49 (2)	41.8 (0.5)	42 (1)	42 (2)	41.3 (0.5)	44.1 (0.1)	39 (1)	51 (2)	46 (1)
Hexanal	1311 (5)	1091 (22)	1190 (17)	640 (3)	835 (6)	1152 (5)	830 (3)	959 (10)	1163.3 (0.1)	609 (5)	1080 (26)	956 (14)	850 (12)	780 (2)	765 (23)	721 (9)	892 (4)	786 (5)	987 (1)	949 (12)
(E)-2-Hexenal	50621 (749)	14474 (99)	38426 (464)	15522 (21)	25969 (193)	41210 (278)	22565 (87)	17189 (152)	29366 (80)	7528 (3)	17220 (109)	9829 (37)	16682 (34)	13761 (21)	6935 (453)	6818 (23)	18713 (6)	16036 (89)	11744 (67)	17241 (24)
(E,E)-2,4-Hexadienal	712 (24)	236 (2)	523 (5)	215 (2)	294 (4)	441 (1)	354.8 (0.3)	375 (2)	506 (1)	149 (1)	308 (6)	278 (1)	313 (3)	268 (1)	224 (7)	208 (3)	409 (3)	324 (4)	348 (2)	407 (2)
Benzaldehyde	34 (1)	54 (2)	37.5 (0.4)	32 (1)	30 (1)	32 (1)	52 (1)	122 (1)	111 (1)	93 (1)	99.1 (0.5)	132 (1)	85 (1)	92.5 (0.4)	126 (5)	124.3 (0.3)	84 (1)	83 (1)	136 (1)	99.5 (0.1)
Sum of aldehydes at C_5 and C_6	52719 (749)	15883 (102)	40222 (465)	16459 (22)	27160 (193)	42876 (278)	23830 (87)	18606 (152)	31119 (80)	8355 (6)	18693 (112)	11147 (39)	17906 (36)	14877 (22)	8003 (454)	7821 (25)	20081 (8)	17214 (89)	13167 (67)	18669 (26)
Heptanal	64 (2)	110 (4)	75 (1)	65 (7)	26 (1)	65 (1)	64 (2)	97 (3)	86 (4)	79 (2)	103 (1)	91 (4)	73 (3)	76 (4)	82 (6)	88 (1)	75 (2)	66.2 (0.4)	99 (2)	80 (3)
(E)-2-Heptenal	69 (2)	306 (3)	125 (3)	74 (3)	58 (1)	72 (1)	97 (1)	171 (3)	149 (1)	136 (4)	258 (2)	229 (2)	117 (4)	109 (4)	79 (8)	n.d.	121.9 (0.5)	110 (1)	194 (1)	148 (2)
(E,E)-2,4-Heptadienal	27.3 (0.5)	62.8 (0.9)	33.2 (0.2)	25.7 (0.1)	71 (1)	26.1 (0.1)	31.4 (0.1)	n.d.	n.d.	n.d.	54.9 (0.3)	n.d.	29.1 (0.3)	29.9 (0.2)	n.d.	45.4 (0.4)	30.1 (0.2)	n.d.	n.d.	n.d.
Octanal	90 (1)	135.6 (0.6)	125 (4)	150.3 (0.1)	149 (3)	121 (3)	89 (4)	163 (1)	120 (2)	99.6 (0.4)	210 (3)	176.2 (0.4)	136 (7)	145 (2)	n.d.	n.d.	135 (1)	108 (2)	98 (5)	108 (7)
(E)-2-Octenal	n.d.	n.d.	n.d.	50 (1)	47 (1)	n.d.	n.d.	n.d.	n.d.	n.d.	n.d.	n.d.	n.d.	n.d.	n.d.	n.d.	n.d.	n.d.	n.d.	n.d.
Nonanal	734 (34)	953 (8)	761 (10)	719 (5)	642 (16)	628 (3)	579 (5)	1407 (82)	1201 (74)	796 (53)	924 (3)	1406 (86)	589 (8)	750 (62)	1173 (31)	1203 (29)	498 (35)	463 (8)	627 (8)	530 (14)
(E)-2-Decenal	101 (3)	204 (7)	176 (1)	207 (11)	204 (8)	154 (8)	110 (4)	417 (7)	161 (5)	162 (8)	225 (15)	492 (7)	119 (10)	123 (7)	239 (1)	218 (17)	108 (8)	259 (1)	201 (10)	111 (10)
(E,E)-2,4-Decadienal	n.d.	n.d.	n.d.	n.d.	n.d.	n.d.	n.d.	n.d.	n.d.	n.d.	n.d.	n.d.	n.d.	n.d.	n.d.	n.d.	n.d.	n.d.	n.d.	n.d.
Sum of aldehydes at C_7 and C_{10}	1084 (34)	1771 (12)	1295 (11)	1291 (14)	1196 (18)	1066 (10)	971 (8)	2255 (82)	1716 (75)	1272 (54)	1774 (15)	2394 (86)	1063 (16)	1233 (63)	1573 (33)	1555 (33)	968 (36)	1006 (9)	1218 (14)	978 (19)
**Alcohols**																				
Ethanol	315 (5)	737 (10)	401 (2)	137 (4)	138 (32)	224 (23)	2093 (26)	1719 (16)	1357 (11)	221 (8)	849 (10)	1800 (15)	908 (1)	631 (10)	1445 (2)	1002 (1)	1616 (1)	2306 (2)	1913 (13)	1449 (13)
1-Pentanol	77.2 (0.1)	85 (3)	308 (2)	103 (2)	95 (1)	57 (2)	85 (4)	85 (1)	83 (1)	82 (2)	86.3 (0.3)	90 (1)	87 (2)	101 (1)	95 (4)	86 (4)	84.0 (0.1)	88 (2)	90.7 (0.1)	84 (1)
1-Penten-3-ol	364 (4)	259 (3)	31.2 (0.4)	269.9 (0.5)	285.6 (2)	337 (2)	294 (0.5)	370 (1)	400 (2)	259 (2)	277 (3)	363.5 (0.1)	260 (4)	281.7 (0.4)	358 (2)	344.4 (0.4)	272 (1)	287 (3)	389 (1)	298 (3)
(E)-2-Penten-1-ol	32.3 (0.1)	36.2 (0.1)	79 (1)	28.2 (0.1)	28.5 (0.1)	33.4 (0.5)	38 (1)	30 (1)	188 (206)	45.4 (0.1)	38.3 (0.3)	34.2 (3)	31.7 (0.1)	33.3 (0.1)	260 (14)	39.2 (0.2)	35.3 (0.3)	38.2 (0.5)	44 (0.4)	36.4 (0.1)
(Z)-2-Penten-1-ol	379 (1)	215 (2)	309 (5)	217 (3)	258 (3)	341 (5)	268.3 (0.4)	354 (1)	393 (2)	190 (3)	227 (2)	332 (5)	202 (5)	208 (4)	306 (16)	292 (2)	215 (1)	232 (1)	348 (2)	249 (2)
1-Hexanol	692 (5)	689 (1)	567 (10)	1038 (11)	876 (13)	813 (12)	1623 (4)	939 (3)	900 (7)	1085 (2)	838 (12)	1001 (9)	1135 (8)	1242 (7)	1050 (59)	1245 (6)	1301 (14)	1759 (7)	1183 (21)	1111.3 (0.4)
(E)-2-Hexen-1-ol	1026 (17)	777 (3)	734 (15)	744 (13)	734 (14)	879 (4)	1771 (17)	697 (10)	794 (1)	965.8 (0.4)	870 (1)	722 (8)	894 (14)	898 (2)	681 (15)	779 (6)	1190 (10)	1808 (6)	807 (4)	870 (7)
(Z)-3-Hexen-1-ol	409 (13)	302 (3)	344 (5)	3928 (48)	2770 (18)	1764 (7)	2261 (24)	1957 (11)	1600 (10)	3908 (33)	847 (4)	1986 (17)	3011 (44)	4039 (15)	2998 (294)	4160 (52)	2449 (7)	2815 (30)	2674 (19)	2373 (3)
1-Heptanol	26 (1)	60 (1)	34.0 (0.4)	35.9 (0.1)	30.2 (0.4)	27.5 (0.4)	35 (0.2)	57.4 (0.2)	49 (1)	43.2 (0.4)	57.6 (0.4)	66.7 (1)	34.7 (0.2)	37.6 (0.5)	56 (6)	54 (1)	33.5 (0.1)	35.2 (0.4)	60.9 (0.3)	41.4 (0.1)
Benzyl alcohol	116 (6)	178 (3)	138 (4)	140 (5)	132 (7)	120 (4)	343 (12)	306 (13)	256 (6)	163 (3)	235 (18)	324 (4)	260 (1)	253 (4)	324 (7)	264 (1)	316 (6)	359 (5)	350 (9)	314 (6)
Phenylethyl Alcohol	330 (8)	265 (3)	340 (8)	234 (13)	255 (8)	291 (15)	410 (6)	1068 (10)	882 (28)	237 (1)	372 (3)	994 (43)	384 (7)	378 (18)	931 (40)	748 (42)	444 (28)	408 (17)	1044 (14)	671 (19)
Sum of alcohols at C_5 and C_7	3005 (22)	2424 (6)	2407 (20)	6364 (51)	5078 (26)	4251 (16)	6376 (30)	4490 (15)	4407 (207)	6579 (34)	3241 (13)	4595 (21)	5655 (47)	6841 (17)	5804 (301)	6999 (53)	5579 (19)	7062 (31)	5597 (29)	5062 (8)
**Esters**																				
Ethyl Acetate	6.3 (0.4)	24.2 (0.2)	11.6 (0.1)	712 (3)	477 (2)	276 (1)	174 (1)	66 (5)	50.9 (0.4)	742 (3)	68.6 (0.4)	70.0 (0.5)	349 (1)	585 (3)	312 (4)	553 (2)	178.2 (0.4)	211 (4)	112 (1)	143.0 (0.4)
Acetic acid, hexyl ester	224 (4)	118.9 (0.4)	180 (1)	169 (5)	177 (6)	214 (2)	451 (2)	2166 (32)	1705 (2)	150.9 (0.5)	252 (3)	2156 (7)	466 (2)	388 (7)	1971 (22)	1574.5 (0.3)	546.9 (0.1)	537 (6)	2383 (9)	1122 (4)
(Z)-3-Hexen-1-ol, acetate	330 (10)	254 (9)	286 (3)	1726 (47)	1267 (36)	877.2 (0.4)	2544 (23)	8668 (91)	6608 (69)	1686 (19)	1269 (9)	8642 (101)	3466 (44)	3283 (28)	7450 (354)	7245 (36)	3684 (11)	3389 (57)	9964 (12)	5712 (61)
Sum of esters at C6	554 (11)	373 (9)	466 (3)	1894 (47)	1444 (37)	1091 (2)	2996 (23)	10834 (97)	8313 (69)	1837 (19)	1521 (10)	10798 (101)	3932 (44)	3671 (28)	9421 (354)	8819 (36)	4231 (11)	3925 (57)	12347 (15)	6834 (61)
**Ketones**																				
3-Pentanone	54 (1)	100 (1)	48.4 (0.4)	309.0 (0.5)	218 (6)	142 (10)	164 (3)	137 (1)	124 (8)	332 (2)	103 (9)	170 (1)	165 (9)	272 (1)	235 (14)	318.9 (0.1)	125 (8)	196 (1)	180 (6)	122 (4)
1-Penten-3-one	204 (3)	215 (3)	193 (2)	80 (1)	113.5 (0.2)	167.3 (0.1)	146.6 (0.5)	201 (2)	215 (2)	85 (1)	215 (2)	218 (1)	148 (3)	120 (1)	159 (18)	137 (1)	172 (4)	145 (1)	217 (2)	193 (2)
5-Hepten-2-one, 6-methyl-	41.3 (0.5)	33 (1)	40 (1)	44 (1)	38.4 (0.4)	38.3 (0.6)	25 (0.2)	92.3 (0.8)	81.0 (0.1)	46.0 (0.4)	32.2 (0.3)	93 (1)	28.9 (0.3)	34.7 (0.2)	88 (5)	82 (1)	24.8 (0.3)	22.5 (0.3)	98.8 (0.4)	49.3 (0.5)
2-Octanone	11.5 (0.1)	n.d.	15 (1)	8.6 (0.4)	8.1 (0.3)	10.6 (0.4)	11.6 (1.1)	22.7 (0.3)	19.4 (0.5)	17.3 (0.5)	36 (2)	35 (2)	19 (2)	12 (1)	23 (2)	20.1 (0.4)	n.d.	10.6 (0.1)	26 (2)	11 (1)
1-Octen-3-one	71 (3)	46 (3)	65.8 (0.2)	63.1 (0.6)	65.2 (1.6)	70 (2)	79 (0.6)	n.d.	n.d.	60 (3)	81 (2)	n.d.	n.d.	n.d.	n.d.	n.d.	n.d.	695 (7)	n.d.	n.d.
Sum of alcohols at C_5 and C_8	382 (4)	393 (4)	362 (3)	504 (1)	444 (6)	428 (11)	426 (4)	453 (2)	439 (9)	540 (3)	466 (10)	516 (3)	361 (10)	439 (2)	505 (23)	558 (1)	322 (9)	1069 (8)	523 (6)	375 (4)
**Carboxylic acids**																				
Acetic acid	151 (7)	423 (1)	222 (4)	34943 (607)	19734 (83)	9886 (46)	2128 (22)	395 (10)	359 (11)	30958 (390)	497 (36)	423 (4)	8365 (140)	19536 (93)	7976 (586)	18423 (62)	941 (34)	2046 (24)	478 (24)	395 (2)
Propanoic acid	13 (2)	71 (8)	69 (1)	114 (6)	100.0 (0.2)	77 (2)	n.d.	66 (3)	n.d.	n.d.	n.d.	n.d.	n.d.	n.d.	n.d.	n.d.	n.d.	n.d.	n.d.	n.d.
Butanoic acid	27 (4)	18 (4)	21 (1)	34 (2)	27.9 (0.2)	22.2 (0.7)	n.d.	n.d.	n.d.	n.d.	n.d.	n.d.	n.d.	n.d.	n.d.	n.d.	n.d.	n.d.	n.d.	n.d.
Hexanoic acid	n.d.	n.d.	n.d.	n.d.	n.d.	n.d.	n.d.	n.d.	n.d.	n.d.	n.d.	n.d.	n.d.	n.d.	n.d.	n.d.	n.d.	n.d.	n.d.	n.d.
Sum of carboxylic acids at C_2 and C_6	191 (8)	512 (9)	313 (4)	35091 (607)	19862 (83)	9984 (46)	2128 (22)	462 (10)	359 (11)	30958 (390)	497 (36)	423 (4)	8365 (140)	19536 (93)	7976 (586)	18423 (62)	941 (34)	2046 (24)	478 (24)	395 (2)
**Hydrocarbons**																				
Octane	68 (1)	100.4 (0.1)	73.6 (0.4)	84.7 (0.1)	78.9 (0.4)	80.3 (0.3)	76 (2)	69.0 (0.5)	60.9 (2.4)	n.d.	53 (2)	75.0 (0.2)	39 (1)	40 (1)	68.4 (0.5)	56.6 (0.1)	44.8 (0.1)	52 (1)	71 (1)	51 (2)

*The results are the means of two independent determinations ± standard deviation. Legend S: sample.
