# Supplementary material for: Potential of the Oxidized Form of the Oleuropein Aglycon to Monitor the Oil Quality Evolution of Commercial Extra-Virgin Olive Oils
Source: Foods. 2023 Aug 4;12(15):2959. doi: 10.3390/foods12152959 (PMC10418756; doi:10.3390/foods12152959)
Supplement: Supplementary file 1 [file foods-12-02959-s001.zip › Table S4.pdf]

Table S4: Composition in antioxidants (mg/kg), value of OSI time (hour), and content of 3-4-DHPEA-EA-OX (mg/kg) of the 20 VOOs at time 0*

	Hydroxytyrosol (3,4-DHPEA)	Tyrosol (*p*-HPEA)	Vanillic acid	Oleacein (3,4-DHPEA-	Oleuropein aglycon 3,4-DHPEA-EA	Oleochantal (*p*-HPEA-EDA)	Ligstroside aglycone (*p*-HPEA-EA)	(+)-1-Acetoxypinoresinol	(+)-Pinoresinol	Sum of the phenolic fractions	α-Tocopherol	OSI	3,4-DHPEA-EA-OX
S1	2.1 (0.1)	2.2 (0.1)	0.22 (0.01)	508.4 (8.0)	134.2 (6.0)	32.34 (0.03)	25 (2.7)	276.9 (19.2)	21.7 (2.7)	1003.2 (29.2)	193.2 (0.3)	33.2 (1.2)	0.94 (0.03)
S2	4.05 (0.01)	3.13 (0.02)	0.042 (0.002)	163.1 (3.8)	36.5 (0.4)	13.824 (0.001)	13.4 (0.1)	206.5 (2.4)	14.6 (0.2)	455.1 (5.1)	360.6 (1.5)	10.7 (0.8)	0.371 (0.001)
S3	2.7 (0.1)	2.49 (0.02)	0.180 (0.001)	387 (1.4)	97.7 (0.3)	26.593 (0.001)	18.09 (0.04)	252.2 (0.9)	18.5 (0.1)	805.3 (1.9)	234.3 (0.4)	26.1 (1.2)	0.809 (0.003)
S4	6.4 (0.1)	3.44 (0.03)	0.370 (0.003)	483.6 (4.9)	71.9 (0.6)	17.769 (0.003)	20.7 (0.1)	352.4 (2.4)	8.5 (10.6)	965.1 (12.2)	192.8 (2.0)	49.3 (2.1)	n.d.
S5	5.36 (0.03)	3.13 (0.01)	0.330 (0.001)	483.7 (0.7)	88.4 (0.1)	21.906 (0.001)	20.4 (0.1)	339 (1.1)	18.5 (0.1)	980.6 (1.7)	185.2 (0.5)	40.6 (1.8)	n.d.
S6	4.16 (0.01)	2.75 (0.01)	0.2731 (0.0003)	477.0 (2.6)	103.7 (0.7)	25.847 (0.004)	19.5 (0.2)	309.4 (2.1)	19.5 (0.1)	962.2 (4.0)	189.5 (0.1)	40.0 (2.0)	n.d.
S7	2.54 (0.01)	2.290 (0.004)	0.26 (0.02)	266.5 (0.8)	52.6 (0.1)	25.537 (0.002)	9.4 (0.1)	104.0 (0.7)	7.4 (0.1)	470.7 (1.3)	170.70 (0.04)	23.3 (1.3)	1.50 (0.06)
S8	7.04 (0.01)	5.78 (0.01)	0.535 (0.004)	191.3 (0.9)	47.49 (0.04)	18.101 (0.004)	9.25 (0.01)	122.5 (0.3)	8.53 (0.02)	410.6 (1.0)	244.4 (1.0)	21.5 (0.6)	1.998 (0.006)
S9	6.27 (0.05)	5.09 (0.04)	0.46 (0.01)	305.5 (3.3)	75.4 (0.7)	23.732 (0.002)	13.1 (0.1)	174.2 (0.6)	12.81 (0.02)	616.6 (3.5)	232.1 (0.3)	25.4 (1.2)	0.781(0.002)
S10	7.58 (0.06)	3.96 (0.04)	0.360 (0.005)	421.1 (3.5)	52.5 (0.3)	14.120 (0.001)	17.9 (0.1)	325.9 (1.7)	14.4 (0.1)	857.8 (4.3)	227.7 (0.6)	36.0 (1.5)	0.65 (0.05)
S11	3.52 (0.01)	2.43 (0.01)	0.060 (0.005)	139.1 (0.5)	24.37 (0.01)	12.651 (0.001)	7.47 (0.01)	122.01 (0.01)	7.7 (0.1)	319.4 (0.5)	357.6 (1.1)	12.6 (0.5)	1.31 (0.02)
S12	4.97 (0.05)	4.2 (0.1)	0.349 (0.004)	74.5 (1.5)	17.8 (0.2)	8.921 (0.003)	4.8 (0.1)	67.1 (1.4)	4.1 (0.3)	186.7 (2.5)	290.0 (0.4)	15.6 (0.8)	0.972 (0.003)
S13	4.469 (0.004)	2.481 (0.003)	0.195 (0.003)	300.2 (0.2)	32.05 (0.05)	24.14 (0.01)	10.186 (0.005)	137.7 (0.3)	6.4 (0.1)	517.9 (0.5)	242.5 (1.1)	20.7 (0.6)	n.d.
S14	6.66 (0.03)	3.41 (0.02)	0.322 (0.004)	429,0 (2.6)	47.2 (0.4)	23.343 (0.004)	14.6 (0.1)	243.8 (1.3)	10.7 (0.2)	778.9 (3.2)	221.8 (0.4)	26.4 (1.5)	0.634 (0.002)
S15	11.3 (0.1)	8.3 (0.1)	0.81 (0.01)	264.3 (2.7)	48.1 (0.6)	20.121 (0.005)	12.5 (0.2)	200.9 (2.2)	10.8 (0.1)	577.0 (4.2)	257.0 (0.5)	24.7 (1.1)	1.699 (0.005)
S16	9.61 (0.01)	6.18 (0.01)	0.60 (0.01)	328.9 (1.0)	47 (0.2)	16.238 (0.001)	12.7 (0.1)	244.8 (0.9)	11.7 (0.2)	677.8 (1.6)	229.8 (1.3)	32.5 (2.0)	1.074 (0.003)
S17	2.52 (0.01)	1.64 (0.03)	0.13 (0.01)	193.1 (0.9)	23.1 (0.1)	22.522 (0.003)	6.07 (0.05)	53.5 (0.5)	2.81 (0.1)	305.4 (1.2)	240.0 (2.6)	15.9 (0.8)	n.d.
S18	2.9 (0.01)	2.36 (0.03)	0.247 (0.005)	223.2 (0.3)	33.1 (0.01)	26.462 (0.001)	7.34 (0.03)	32.5 (0.1)	3.5 (0.1)	331.5 (0.3)	192.4 (0.5)	17.6 (0.8)	n.d.
S19	5.464 (0.002)	4.563 (0.003)	0.43 (0.01)	104.7 (0.2)	21.1 (0.1)	13.029 (0.002)	5.22 (0.03)	62.3 (0.6)	3.9 (0.1)	220.6 (0.8)	258.8 (10.9)	16.8 (0.9)	1.201 (0.004)
S20	3.248 (0.001)	2.42 (0.02)	0.201 (0.005)	133.6 (1.1)	17.9 (0.01)	15.853 (0.001)	4.80 (0.01)	22.46 (0.01)	2.6 (0.1)	203.1 (1.1)	244.4 (0.7)	16.6 (1.1)	0.519 (0.002)

*The results are the means of two independent de-terminations ± standard deviation. Legend S: sample; OSI: Oxidative Stability Index; 3-4-DPEA-EA-OX: Oxidized form of 3,4-DHPE-EA.
