# Supplementary material for: Potential of the Oxidized Form of the Oleuropein Aglycon to Monitor the Oil Quality Evolution of Commercial Extra-Virgin Olive Oils
Source: Foods. 2023 Aug 4;12(15):2959. doi: 10.3390/foods12152959 (PMC10418756; doi:10.3390/foods12152959)
Supplement: Supplementary file 1 [file foods-12-02959-s001.zip › Table S5.pdf]

Table S5: Evolution of the extinction coefficient K_{270} over 12 month storage with light exposure in VOOlmp and VOOmhp samples*

Time (months)		0	1	2	3	4	5	6	7	8	9	10	11	12
VOOlmp	S13	0.115 (0.003) a	0.174 (0.005) b	0.197 (0.006) bc	0.199 (0.006) c	0.201 (0.006) c	0.200 (0.006) c	0.211 (0.006) cde	0.229 (0.006) defg	0.209 (0.006) cg	0.233 (0.007) ef	0.233 (0.007) ef	0.235 (0.007) f	0.248 (0.007) f
	S7	0.106 (0.003) a	0.141 (0.004) b	0.146 (0.004) bc	0.165 (0.005) cd	0.166 (0.005) d	0.168 (0.005) d	0.169 (0.005) d	0.174 (0.005) d	0.178 (0.005) d	0.198 (0.006) e	0.198 (0.006) e	0.199 (0.006) e	0.205 (0.006) e
	S2	0.129 (0.004) a	0.211 (0.006) b	0.215 (0.006) b	0.220 (0.006) b	0.221 (0.006) b	0.226 (0.006) bc	0.225 (0.006) bc	0.247 (0.007) cd	0.248 (0.007) cd	0.254 (0.007) d	0.265 (0.007) d	0.265 (0.007) d	0.267 (0.007) d
	S8	0.133 (0.004) a	0.171 (0.005) b	0.177 (0.005) b	0.180 (0.005) b	0.187 (0.005) b	0.189 (0.005) bd	0.211 (0.006) de	0.211 (0.006) de	0.215 (0.006) e	0.232 (0.006) efg	0.241 (0.007) fgh	0.221 (0.006) eh	0.244 (0.007) g
	S18	0.094 (0.003) a	0.136 (0.004) b	0.153 (0.004) bc	0.169 (0.005) cd	0.168 (0.005) cd	0.170 (0.005) cd	0.175 (0.005) de	0.19 (0.005) efg	0.178 (0.005) deg	0.190 (0.005) ef	0.194 (0.005) ef	0.193 (0.005) ef	0.207 (0.006) f
	S11	0.124 (0.003) a	0.189 (0.005) b	0.195 (0.005) b	0.212 (0.006) bc	0.213 (0.006) bcd	0.223 (0.006) cd	0.238 (0.007) def	0.236 (0.007) cef	0.233 (0.007) ce	0.260 (0.007) fg	0.264 (0.007) g	0.264 (0.007) g	0.270 (0.008) g
	S17	0.109 (0.003) a	0.163 (0.005) b	0.181 (0.005) b	0.208 (0.006) c	0.206 (0.006) c	0.209 (0.006) c	0.210 (0.006) c	0.221 (0.006) ce	0.212 (0.006) ce	0.216 (0.006) ce	0.223 (0.006) ce	0.224 (0.006) ce	0.234 (0.007) de
	S19	0.125 (0.004) a	0.188 (0.005) b	0.198 (0.006) bc	0.212 (0.006) bce	0.213 (0.006) bce	0.212 (0.006) bce	0.223 (0.006) cdef	0.247 (0.007) defgh	0.237 (0.007) efg	0.247 (0.007) fgh	0.250 (0.007) gh	0.264 (0.007) h	0.271 (0.008) h
	S20	0.120 (0.003) a	0.181 (0.005) b	0.204 (0.006) bc	0.214 (0.006) cd	0.208 (0.006) ce	0.216 (0.006) cd	0.226 (0.006) cd	0.230 (0.006) de	0.234 (0.007) d	0.263 (0.007) f	0.265 (0.007) f	0.277 (0.008) f	0.285 (0.008) f
	S12	0.125 (0.004) a	0.180 (0.005) b	0.219 (0.006) c	0.226 (0.006) c	0.225 (0.006) c	0.227 (0.006) cd	0.241 (0.007) cde	0.253 (0.007) def	0.258 (0.007) efg	0.271 (0.008) fgh	0.272 (0.008) fgh	0.281 (0.008) gh	0.293 (0.008) h
VOOmhp	S1	0.134 (0.003) a	0.186 (0.006) b	0.190 (0.009) bcd	0.197 (0.007) bcdn	0.218 (0.009) cdefghil	0.211 (0.006) bedfghi	0.221 (0.009) dfghilm	0.233 (0.007) fghilm	0.224 (0.008) ghilmn	0.233 (0.006) hilm	0.241 (0.011) ilm	0.246 (0.009) lm	0.250 (0.009) m
	S5	0.125 (0.002) a	0.172 (0.006) b	0.173 (0.005) b	0.175 (0.005) b	0.178 (0.005) b	0.181 (0.006) b	0.193 (0.006) b	0.194 (0.005) b	0.207 (0.006) cd	0.209 (0.006) cd	0.209 (0.007) cd	0.205 (0.006) cd	0.222 (0.007) d
	S4	0.132 (0.003) a	0.147 (0.006) b	0.172 (0.008) bce	0.179 (0.007) cde	0.183 (0.005) cde	0.178 (0.008) cde	0.187 (0.007) cde	0.205 (0.007) defg	0.188 (0.007) eh	0.219 (0.007) fg	0.215 (0.007) fhg	0.215 (0.008) fhg	0.218 (0.009) g
	S6	0.132 (0.005) a	0.172 (0.007) be	0.175 (0.008) be	0.177 (0.006) bce	0.190 (0.005) bcde	0.183 (0.007) bce	0.205 (0.007) cdef	0.213 (0.006) def	0.191 (0.005) e	0.225 (0.007) f	0.226 (0.01) f	0.228 (0.008) f	0.229 (0.009) f
	S10	0.118 (0.005) a	0.172 (0.007) b	0.183 (0.008) bce	0.193 (0.006) bce	0.197 (0.005) bce	0.198 (0.007) bce	0.202 (0.007) ce	0.234 (0.007) dfg	0.209 (0.005) efg	0.232 (0.007) f	0.234 (0.011) f	0.233 (0.008) f	0.236 (0.009) f
	S3	0.132 (0.004) a	0.187 (0.007) b	0.201 (0.008) b	0.203 (0.007) b	0.206 (0.005) bc	0.208 (0.008) bc	0.235 (0.008) cd	0.243 (0.007) d	0.246 (0.006) d	0.250 (0.008) d	0.250 (0.011) d	0.253 (0.008) d	0.262 (0.01) d
	S14	0.113 (0.003) a	0.167 (0.006) b	0.181 (0.007) bc	0.195 (0.006) ceh	0.194 (0.004) ceh	0.191 (0.007) bch	0.206 (0.007) cdef	0.229 (0.007) dfgi	0.204 (0.005) cfi	0.218 (0.007) efgi	0.218 (0.009) efgi	0.224 (0.007) fg	0.233 (0.009) g
	S16	0.122 (0.005) a	0.167 (0.006) b	0.180 (0.007) bce	0.180 (0.006) bce	0.189 (0.008) bce	0.194 (0.007) bce	0.206 (0.007) cde	0.224 (0.008) dfg	0.206 (0.007) eg	0.236 (0.007) f	0.236 (0.009) f	0.238 (0.007) f	0.250(0.009) f
	S9	0.133 (0.004) a	0.174 (0.006) b	0.175 (0.007) b	0.176 (0.005) b	0.190 (0.004) bc	0.192 (0.007) bc	0.199 (0.006) bc	0.207 (0.006) c	0.203 (0.007) c	0.235 (0.009) d	0.238 (0.008) d	0.243 (0.007) d	0.251 (0.009) d
	S15	0.125 (0.004) a	0.171 (0.005) b	0.196 (0.007) bcd	0.201 (0.007) cde	0.199 (0.007) bde	0.202 (0.006) d	0.217 (0.007) df	0.236 (0.008) fg	0.238 (0.007) fgh	0.240 (0.009) fgh	0.254 (0.008) gh	0.256 (0.008) gh	0.266 (0.008) h

*The results are the means of two independent determinations ± standard deviation. Different letters in each row indicate statistically different values at $p < 0.05$. Legend: VOOlmp: Virgin olive oil with low-medium polyphenol content; VOOmhp: Virgin olive oil with medium-high polyphenol content.
