# Supplementary material for: Potential of the Oxidized Form of the Oleuropein Aglycon to Monitor the Oil Quality Evolution of Commercial Extra-Virgin Olive Oils
Source: Foods. 2023 Aug 4;12(15):2959. doi: 10.3390/foods12152959 (PMC10418756; doi:10.3390/foods12152959)
Supplement: Supplementary file 1 [file foods-12-02959-s001.zip › Table S6.pdf]

Table S6: Evolution of the extinction coefficient ΔK over 12 month storage with light exposure in VOOlmp and VOOmhp samples*

Time (months)		0	1	2	3	4	5	6	7	8	9	10	11	12
VOOlmhp	S13	-0.0050 (0.0002) a	0.0065 (0.0002) b	0.0080 (0.0003) cg	0.0055 (0.0002) b	0.0095 (0.0004) defl	0.0080 (0.0003) cg	0.0105 (0.0004) efhl	0.0095 (0.0003) fml	0.0090 (0.0004) dgmn	0.0110 (0.0003) hio	0.0120 (0.0004) i	0.0095 (0.0004) ln	0.0105 (0.0005) lo
	S7	-0.0020 (0.0001) a	0.0040 (0.0001) b	0.0045 (0.0002) b	0.0040 (0.0001) b	0.0060 (0.0002) cd	0.0040 (0.0002) b	0.0060 (0.0002) d	0.0085 (0.0003) e	0.0065 (0.0003) dg	0.0075 (0.0003) f	0.0090 (0.0004) e	0.0070 (0.0003) fg	0.0075 (0.0003) f
	S2	-0.0020 (0.00005) a	0.0095 (0.0004) b	-0.0015 (0.0001) a	0.0095 (0.0004) b	0.0100 (0.0004) b	0.0095 (0.0002) b	0.0115 (0.0004) c	0.0125 (0.0005) cd	0.0125 (0.0004) cd	0.0130 (0.0006) de	0.0130 (0.0004) de	0.0120 (0.0004) ce	0.0120 (0.0003) ce
	S8	-0.0045 (0.0002) a	0.0040 (0.0002) bd	-0.0145 (0.0005) c	0.0045 (0.0002) bd	0.0050 (0.0002) bf	0.0035 (0.0001) d	0.0080 (0.0003) egi	0.0090 (0.0003) egim	0.0060 (0.0003) f	0.0085 (0.0003) gl	0.0110 (0.0004) h	0.0100 (0.0004) hmn	0.0090 (0.0003) iln
	S18	-0.0030 (0.0001) a	0.0055 (0.0002) b	0.0055 (0.0002) b	0.0065 (0.0002) bd	0.0060 (0.0003) b	0.0060 (0.0002) b	0.0090 (0.0003) cf	0.0080 (0.0002) cg	0.0075 (0.0003) dg	0.0105 (0.0004) e	0.0105 (0.0004) e	0.0090 (0.0003) cf	0.0095 (0.0003) ef
	S11	-0.0025 (0.0001) a	0.0085 (0.0003) b	-0.0130 (0.0004) c	0.0095 (0.0003) bd	0.0100 (0.0003) d	0.0105 (0.0004) d	0.0130 (0.0004) efgh	0.0135 (0.0004) efgh	0.0125 (0.0004) e	0.0140 (0.0004) fg	0.0140 (0.0004) gh	0.0125 (0.0003) e	0.0140 (0.0004) fh
	S17	-0.0050 (0.0002) a	0.0065 (0.0002) bc	0.0060 (0.0002) b	0.0055 (0.0002) b	0.0075 (0.0002) cd	0.0080 (0.0003) d	0.0100 (0.0004) efh	0.0085 (0.0002) di	0.0085 (0.0002) di	0.0105 (0.0004) fh	0.0120 (0.0005) g	0.0095 (0.0004) fih	0.0105 (0.0003) h
	S19	-0.0050 (0.0001) a	0.0080 (0.0003) b	0.0060 (0.0002) cd	0.0060 (0.0002) d	0.0080 (0.0002) b	0.0075 (0.0003) bcd	0.0110 (0.0004) e	0.0125 (0.0005) efh	0.0115 (0.0005) eh	0.0135 0(0.0006) fgi	0.0145 (0.0004) gi	0.0110 (0.0004) e	0.0130 (0.0005) hi
	S20	-0.0055 (0.0002) a	0.0085 (0.0003) b	0.0090 (0.0003) b	0.0080 (0.0002) b	0.0090 (0.0003) b	0.0090 (0.0003) b	0.0115 (0.0005) cdeh	0.0125 (0.0004) dfhi	0.0105 (0.0004) e	0.0135 (0.0003) fgi	0.0145 (0.0005) g	0.0120 (0.0004) hl	0.0130 (0.0005) il
	S12	-0.0050 (0.0002) a	0.0070 (0.0003) b	0.0085 (0.0003) bc	0.0085 (0.0003) bc	0.0090 (0.0003) c	0.0085 (0.0004) bc	0.0130 (0.0004) degh	0.0125 (0.0006) deg	0.0120 (0.0005) dg	0.0140 (0.0005) efgh	0.0150 (0.0006) fh	0.0125 (0.0005) g	0.0145 (0.0004) h
VOOmhp	S1	-0.0035 (0.0001) a	0.0050 (0.0003) b	-0.0190 (0.0004) c	0.0050 (0.0002) b	0.0065 (0.0001) def	0.0055 (0.0002) bde	0.0065 (0.0002) ef	0.0009 (0.0003) gl	0.0075 (0.0004) fh	0.0090 (0.0003) gi	0.0100 (0.0003) g	0.0080 (0.0003) hil	0.0080 (0.0004) hil
	S5	-0.0045 (0.0001) a	0.0030 (0.0001) b	0.0035 (0.0002) b	0.0035 (0.0001) b	0.0030 (0.0001) b	0.0003 (0.0001) b	0.0055 (0.0002) cdeh	0.0060 (0.0002) dfhi	0.0050 (0.0002) e	0.0065 (0.0003) fl	0.0080 (0.0003) g	0.0060 (0.0002) hl	0.0065 (0.0002) il
	S4	-0.0040 (0.0001) a	0.0035 (0.0001) bc	0.0030 (0.0001) c	0.0040 (0.0001) bf	0.0050 (0.0001) d	0.0040 (0.0001) bf	0.0050 (0.0002) d	0.0065 (0.0002) e	0.0045 (0.0002) df	0.0070 (0.0002) e	0.0095 (0.0004) g	0.0050 (0.0002) d	0.0070 (0.0002) e
	S6	-0.0035 (0.0002) a	0.0030 (0.0001) b	-0.0020 (0.0001) c	0.0040 (0.0002) de	0.0045 (0.0002) d	0.0035 (0.0001) be	0.0060 (0.0002) f	0.0070 (0.0003) g	0.0045 (0.0002) d	0.0075 (0.0002) gh	0.0080 (0.0003) h	0.0055 (0.0002) f	0.0071 (0.0002) g
	S10	-0.0040 (0.0002) a	0.0050 (0.0002) b	0.0060 (0.0002) bc	0.0065 (0.0002) cdeh	0.0075 (0.0003) df	0.0060 (0.0002) be	0.0080 (0.0003) fi	0.0105 (0.0004) g	0.0075 (0.0002) hf	0.0100 (0.0004) gi	0.0105 (0.0004) g	0.0090 (0.0003) i	0.0105 (0.0004) g
	S3	-0.0030 (0.0001) a	0.0050 (0.0002) b	0.0065 (0.0002) c	0.0065 (0.0002) c	0.0075 (0.0003) ce	0.0065 (0.0002) c	0.0090 (0.0003) df	0.0095 (0.0003) d	0.0080 (0.0002) ef	0.0100 (0.0003) dh	0.0110 (0.0003) h	0.010 (0.0004) dh	0.0100 (0.0003) dh
	S14	-0.0055 (0.0003) a	0.0050 (0.0002) b	0.0055 (0.0002) b	0.0050 (0.0002) b	0.0060 (0.0002) bd	0.0060 (0.0001) bd	0.0091 (0.0003) chlm	0.0080 (0.0003) chlmn	0.0070 (0.0002) dn	0.0085 (0.0004) hlm	0.0110 (0.0004) i	0.0080 (0.0003) lno	0.0090 (0.0003) mo
	S16	-0.0055 (0.0002) a	0.0045 (0.0001) bd	0.0060 (0.0002) cf	0.0040 (0.0001) b	0.0055 (0.0002) c	0.0050 (0.0002) d	0.0080 (0.0003) e	0.0085 (0.0003) e	0.0065 (0.0002) f	0.0105 (0.0003) ghl	0.0110 (0.0004) h	0.0085 (0.0003) e	0.0100 (0.0003) l
	S9	-0.0045 (0.0002) a	0.0045 (0.0004) bd	-0.0095 (0.0003) c	0.0040 (0.0001) b	0.0055 (0.0002) de	0.0040 (0.0002) b	0.0060 (0.0002) e	0.0080 (0.0003) f	0.0050 (0.0001) be	0.0090 (0.0003) f	0.0090 (0.0003) f	0.0085 (0.0003) f	0.0090 (0.0003) f
	S15	-0.0050 (0.0002) a	0.0045 (0.0002) b	0.0070 (0.0001) cf	0.0050 (0.0002) bd	0.0060 (0.0003) cdf	0.0060 (0.0002) cdf	0.0095 (0.0003) egi	0.0085 (0.0003) ei	0.0070 (0.0003) f	0.0100 (0.0004) gi	0.0115 (0.0004) h	0.0095 (0.0003) i	0.0125 (0.0006) h

*The results are the means of two independent determinations ± standard deviation. Different letters in each row indicate statistically different values at $p < 0.05$. Legend: VOOlmp: Virgin olive oil with low-medium polyphenol content; VOOmhp: Virgin olive oil with medium-high polyphenol content.
