# Supplementary material for: Potential of the Oxidized Form of the Oleuropein Aglycon to Monitor the Oil Quality Evolution of Commercial Extra-Virgin Olive Oils
Source: Foods. 2023 Aug 4;12(15):2959. doi: 10.3390/foods12152959 (PMC10418756; doi:10.3390/foods12152959)
Supplement: Supplementary file 1 [file foods-12-02959-s001.zip › Table S7.pdf]

Table S7: Evolution of (*E*, *E*)-2,4-decadienal (μg/kg) over 12 month storage with light exposure in VOOlmp and VOOmhp samples*

Time (months)		0	1	2	3	4	5	6	7	8	9	10	11	12
VOOlmp	S13	n.d.	n.d.	n.d.	n.d.	n.d.	n.d.	n.d.	n.d.	99 (2) a	249 (5) b	292 (6) bc	374 (5) cd	416 (35) d
	S7	n.d.	n.d.	n.d.	n.d.	252 (7) a	292 (10) ab	306 (5) b	383.1 (0.3) c	408 (5) c	465 (1) d	487 (4) d	491 (5) d	496 (24) d
	S2	n.d.	n.d.	n.d.	n.d.	331 (1) a	385 (2) a	356 (24) a	481 (11) b	592 (28) c	754 (5) d	790 (8) de	810 (3) de	840 (16) e
	S8	n.d.	n.d.	n.d.	n.d.	n.d.	154 (2) a	284 (6) b	374 (11) c	495 (14) d	584 (26) ef	607 (2) eg	628.9 (0.4) fg	651 (7) g
	S18	n.d.	n.d.	n.d.	n.d.	n.d.	n.d.	n.d.	152 (3) a	163 (3) a	228 (6) b	293.7 (0.3) c	324 (8) d	417 (5) e
	S11	n.d.	n.d.	n.d.	n.d.	162 (9) a	222 (14) ab	232 (9) ac	252 (13) bc	266.2 (0.1) bcde	301 (1) cde	328 (4) de	356 (20) e	366 (23) e
	S17	n.d.	n.d.	n.d.	n.d.	n.d.	n.d.	n.d.	87 (4) a	200 (8) b	246.0 (0.4) c	270 (3) c	310 (2) d	401 (4) e
	S19	n.d.	n.d.	n.d.	n.d.	103 (3) a	114.7 (0.2) a	249 (1) b	297 (4) c	303 (1) c	375 (7) d	402 (3) e	471 (3) f	534 (8) g
	S20	n.d.	n.d.	n.d.	n.d.	n.d.	106 (4) a	192 (2) b	212.9 (0.1) b	245 (2) c	291 (1) d	304 (2) d	339 (11) e	420 (2) f
	S12	n.d.	n.d.	n.d.	n.d.	89 (8) a	103 (9) a	211 (4) b	227 (4) bc	252 (2) bc	256 (10) c	268 (2) c	317 (8) e	396 (15) f
VOOmhp	S1	n.d.	n.d.	n.d.	n.d.	n.d.	n.d.	n.d.	n.d.	n.d.	n.d.	n.d.	453 (21) a	488 (14) a
	S5	n.d.	n.d.	n.d.	n.d.	306 (10) a	314 (12) ab	321 (3) ab	336 (13) ab	355 (11) bc	360 (2) bc	383 (2) c	397 (4) cd	441 (9) d
	S4	n.d.	n.d.	n.d.	n.d.	154 (3) a	168 (1) ab	184 (9) abc	184 (6) abc	186 (8) abc	182 (6) abc	185 (1) abc	198 (9) bc	208 (11) c
	S6	n.d.	n.d.	n.d.	n.d.	n.d.	n.d.	n.d.	n.d.	176 (8) a	180 (3) a	188.7 (0.1) a	237 (4) b	269 (14) b
	S10	n.d.	n.d.	n.d.	n.d.	187 (8) a	197 (8) be	290 (2) cd	305 (9) cf	251 (11) de	240 (4) e	291 (5) cdf	305.3 (0.4) cf	339 (17) f
	S3	n.d.	n.d.	n.d.	n.d.	233 (6) a	291 (8) b	331 (8) bc	344 (4) cd	362 (3) cde	363 (21) cde	392 (2) de	406 (3) e	410 (5) e
	S14	n.d.	n.d.	n.d.	n.d.	95 (1) a	113 (1) ab	152 (10) b	204 (3) c	211 (7) c	240 (10) cd	263 (5) de	296 (11) ef	322 (10) f
	S16	n.d.	n.d.	n.d.	n.d.	n.d.	n.d.	n.d.	n.d.	96 (1) a	215 (3) b	278 (7) c	429 (6) d	502 (4) e
	S9	n.d.	n.d.	n.d.	n.d.	82 (2) a	78 (1) a	129 (4) b	163 (2) c	164 (1) c	179 (9) c	232 (9) d	332 (4) e	446 (3) f
	S15	n.d.	n.d.	n.d.	n.d.	8.0 (0.4) a	15 (8) a	101 (7) bc	119.2 (0.4) cd	139 (2) de	155.9 (0.4) e	191 (3) f	284.7 (0.2) g	318 (7) h

*The results are the means of two independent determinations ± standard deviation. Different letters in each row indicate statistically different values at $p < 0.05$. Legend: VOOlmp: Virgin olive oil with low-medium polyphenol content; VOOmhp: Virgin olive oil with medium-high polyphenol content. N.d.: not detected.
