# Supplementary material for: Potential of the Oxidized Form of the Oleuropein Aglycon to Monitor the Oil Quality Evolution of Commercial Extra-Virgin Olive Oils
Source: Foods. 2023 Aug 4;12(15):2959. doi: 10.3390/foods12152959 (PMC10418756; doi:10.3390/foods12152959)
Supplement: Supplementary file 1 [file foods-12-02959-s001.zip › Table S8.pdf]

Table S8: Evolution of (*E*)-2-decenal (μg/kg) over 12 month storage with light expo-sure in VOOlmp and VOOmhp samples*

Time (months)		0	1	2	3	4	5	6	7	8	9	10	11	12
VOOlmp	S13	119 (10) a	178 (5) b	200 (2) b	290 (5) c	304 (10) c	329 (9) e	362 (4) f	409 (3) g	414 (11) g	440 (9) gh	463 (1) h	504 (9) i	559 (16) l
	S7	110 (4) a	145 (8) ab	172 (10) bc	184 (14) bc	196 (8) c	207 (10) c	258 (10) d	316 (10) e	410 (5) g	483 (11) h	492 (11) h	512 (11) h	525 (26) h
	S2	204 (7) a	218 (7) a	411 (7) b	545 (12) c	589 (6) d	607 (5) de	638 (11) e	780 (5) f	892 (6) g	993 (3) h	1010 (3) h	1043 (14) i	1116 (8) l
	S8	417 (7) a	434 (10) b	494 (11) c	546 (16) c	564.3 (0.2) d	670 (2) e	788 (7) f	869 (3) g	928 (3) gh	961 (4) hi	981 (3) il	1005 (0.1) l	1038 (19) m
	S18	259 (1) a	262 (6) a	286 (4) ab	293 (19) ab	306 (1) b	310 (7) b	303 (1) b	378 (21) c	386 (6) c	475 (10) d	501 (9) d	593 (13) e	654 (6) f
	S11	225 (15) a	259 (11) a	301 (16) b	512 (7) c	534.1 (0.1) c	623 (3) d	658 (21) d	709 (6) e	728 (3) e	783 (2) f	791 (4) f	853 (14) g	915 (7) h
	S17	108 (8) a	145 (6) b	211 (5) c	288 (14) d	293 (4) d	294 (10) d	303 (11) d	356 (9) e	381 (6) e	441 (3) f	450 (3) f	499 (11) h	528 (8) h
	S19	201 (10) a	294 (13) b	281 (7) b	384 (7) c	409 (5) cd	436 (3) d	472 (6) e	492 (5) ef	519 (2) f	584 (4) g	607 (1) g	654 (1) h	686 (13) i
	S20	111 (10) a	125 (8) a	195 (10) b	228 (3) c	234 (8) c	250 (6) c	345 (7) d	454 (1) e	511 (13) f	614 (8) g	657 (9) h	689 (4) i	711 (2) i
	S12	492 (7) a	513 (9) a	641 (10) b	770 (7) c	805 (3) d	897 (5) e	932 (11) f	953 (0) fg	970 (1) gh	988 (4) h	1066 (4) i	1139 (9) l	1162 (12) l
VOOmhp	S1	101 (3) a	170.5 (0.5) b	200 (1) c	288 (4) d	297 (2) d	307 (6) d	337 (17) e	349 (9) ef	354 (3) ef	361 (4) fg	369 (2) fg	381 (2) g	444.0 (0.4) h
	S5	204 (8) a	261 (6) b	275 (10) bc	283 (5) bc	289 (9) cd	308 (4) de	316 (7) e	323 (2) ef	330 (6) efg	341 (5) fg	349 (2) gh	362 (4) h	403 (2) i
	S4	207 (11) a	232 (9) a	277 (12) b	316 (9) c	328 (2) c	341 (3) c	377 (11) d	387.1 (0.4) de	390 (6) de	382 (2) de	394 (2) de	411 (8) e	448 (15) f
	S6	154 (8) a	187 (9) b	195 (3) c	232 (2) d	242.5 (0.2) d	247 (2) d	284 (15) e	296 (3) e	306 (1) ef	327 (4) fg	340 (3) gh	362.2 (0.5) hi	381 (6) i
	S10	162 (8) a	154 (1) b	189 (4) c	189.2 (5.2) c	205 (1) c	286 (4) d	328 (8) e	434 (3) f	510 (1) g	593 (5) h	606 (4) hi	622 (1) il	635 (13) l
	S3	176 (1) a	185 (8) a	245 (3) b	310.8 (3.7) c	326 (1) c	361 (5) d	370 (11) d	380 (3) de	393 (5) ef	400 (3) ef	406 (1) f	444 (1) g	485 (8) h
	S14	123 (7) a	208 (2) b	242 (4) c	293 (2) d	305 (5) d	351.9 (0.1) e	405 (1) f	490 (1) g	557 (6) h	570 (2) h	601.5 (0.4) i	676 (4) l	710 (7) m
	S16	218 (17) a	258 (9) a	310 (9) b	469 (8) c	468 (2) c	470.8 (0.4) c	477 (9) c	480 (4) c	484 (3) c	494 (3) c	503 (5) c	623 (14) d	711 (25) e
	S9	161 (5) a	179 (7) ab	206 (6) bc	227 (14) cd	234 (2) d	240.2 (0.4) d	268 (9) e	369 (4) f	378 (3) f	391 (3) f	421 (5) g	474 (3) h	541 (11) i
	S15	239 (1) a	272 (4) b	385 (2) c	503 (3) d	518 (1) e	530 (3) ef	543.1 (0.1) f	586.1 (0.5) g	604 (1) h	621 (3) i	672 (5) l	799 (6) m	942 (6) n

*The results are the means of two independent determinations ± standard deviation. Different letters in each row indicate statistically different values at $p < 0.05$. Legend: VOOlmp: Virgin olive oil with low-medium polyphenol content; VOOmhp: Virgin olive oil with medium-high polyphenol content. N.d.: Not detected.
