# Supplementary material for: Potential of the Oxidized Form of the Oleuropein Aglycon to Monitor the Oil Quality Evolution of Commercial Extra-Virgin Olive Oils
Source: Foods. 2023 Aug 4;12(15):2959. doi: 10.3390/foods12152959 (PMC10418756; doi:10.3390/foods12152959)
Supplement: Supplementary file 1 [file foods-12-02959-s001.zip › Table S9.pdf]

Table S9: Evolution of the sum* of the phenolic fractions over 12month storage with light exposure in VOOlmp and VOOmhp samples**

Time (months)		0	1	2	3	4	5	6	7	8	9	10	11	12
VOOlmp	S13	517.9 (0.5) a	499.7 (3.3) b	464.8 (3.8) c	428.6 (2.7) d	406.1 (2.5) e	381.9 (8.0) f	356.4 (6.1) g	309.1 (2.3) h	294.7 (7.7) h	259.2 (6.3) i	208.0 (4.2) l	88.8 (0.1) m	67.8 (2.2) n
	S7	470.7 (1.3) a	447.6 (13.3) b	433.3 (3.1) b	393.3 (6.9) c	361.8 (2.9) d	351.9 (5.8) de	334.2 (3.2) e	303.8 (5.4) f	242.9 (8.0) g	159.8 (5.7) h	136.9 (2.4) hi	116.0 (0.2) i	109.6 (3.0) i
	S2	455.1 (5.1) a	365 (2.8) b	325.8 (3.7) c	277 (13.3) d	246.2 (2.9) e	232.9 (0.2) ef	226.6 (4.0) fg	210.0 (2.7) g	172.4 (3.9) h	135.3 (4.3) i	105.2 (3.4) l	78.5 (0.1) m	68.1 (1.1) m
	S8	410.6 (1.0) a	357.9 (5.7) b	334.5 (10.1) c	298.3 (3) d	283.1 (2.9) de	272.5 (2.6) e	253.4 (5.8) f	231.4 (4.0) g	198.3 (5.2) h	153.6 (3.4) i	130.4 (5.2) l	100.5 (0.1) m	78.6 (1.3) n
	S18	331.5 (0.3) a	316.7 (2.6) b	290.2 (1.4) c	269.9 (2.5) d	258.7 (2.4) e	223.2 (1.7) f	210.8 (2.6) g	201.9 (3.9) g	175.4 (2.2) h	139.9 (0.8) i	113.0 (3.7) l	92.1 (2.1) m	77.0 (1.5) n
	S11	319.4 (0.5) a	306.0 (9.4) a	276.8 (4.7) b	244.9 (3.9) c	238.0 (1.1) c	213.6 (2.8) d	171.5 (3.8) e	135.2 (2.0) f	107.5 (3.0) g	69.4 (4.6) h	58.9 (5.3) hi	50.5 (0.1) il	41.9 (1.6) l
	S17	305.4 (1.2) a	288.4 (1.6) b	271.1 (9.9) c	252.3 (2.1) d	244.5 (0.9) d	217.2 (1.2) e	213.9 (2.1) e	190.5 (8.7) f	171.7 (0.2) g	148.9 (1.1) h	89.6 (0.3) i	67.5 (1.4) l	51.7 (1.1) l
	S19	220.6 (0.8) a	214.6 (2.6) ab	216.4 (3.8) a	209.1 (10.8) ab	195.7 (6.9) bd	187.6 (1.5) cd	179.3 (4.8) d	155.1 (6.2) e	143.5 (1.1) e	91.7 (3.5) f	77.6 (0.6) f	56.7 (0.8) h	50.7 (2.4) h
	S20	203.1 (1.1) a	192.3 (1.4) a	168.7 (8) b	146.0 (2.3) c	131.8 (7.8) cd	120.0 (1.0) d	102.6 (4.8) e	87.3 (2.2) ef	85.5 (2.9) f	74.3 (0.6) f	55.9 (3.9) g	42.8 (0.4) g	39.8 (3.1) g
	S12	186.7 (2.5) a	175.6 (1.6) ab	167.2 (1.9) b	158.3 (2.1) b	140.8 (13) c	118.9 (3.9) d	96.1 (2.4) e	60.1 (0.8) f	47.9 (3.4) fg	44.0 (0.5) fg	41.7 (2.7) g	39.4 (0.1) g	32.2 (2.2) g
VOOmhp	S1	1003.2 (29.2) a	917.3 (7.8) b	781.2 (0.9) c	731.9 (22.4) d	653.2 (9.3) e	632.8 (4.6) e	626.7 (7.2) e	608.2 (3.7) e	520.8 (3.2) f	473.0 (4.0) h	439.4 (3.7) h	388.7 (5.0) i	342.8 (6.6) l
	S5	980.6 (1.7) a	918.3 (13.4) b	846.6 (13.0) c	815.3 (10.6) cd	789.6 (7.7) de	777.3 (11.4) ef	741.4 (5.3) fg	727.6 (18.8) g	710.6 (1.3) gh	687.6 (9.3) h	578.9 (1.9) i	476.5 (2.3) l	350.4 (5.9) m
	S4	965.1 (12.2) a	949.0 (8.4) a	829.2 (3.8) b	822.6 (12.2) bc	795.0 (3.4) c	762.7 (5.6) d	742.3 (6.9) de	724.7 (8.1) ef	709.0 (9.0) f	668.0 (2.1) g	608.9 (5.8) h	517.0 (0.7) i	374.3 (7.0) l
	S6	962.2 (4.0) a	891.2 (9.2) b	831.4 (5.3) c	782.7 (14.0) d	796.2 (7.1) d	776.3 (5.2) d	744.8 (3.5) e	731.4 (5.6) e	716.7 (14.6) ef	691.7 (4.1) f	603.6 (0.6) g	519.4 (7.9) h	412.8 (3.8) i
	S10	857.8 (4.3) a	781.4 (8.5) b	730.2 (0.3) c	622.6 (3.8) d	574.3 (3.2) e	544.2 (3.5) f	502.2 (10.0) g	445.8 (5.5) h	399.9 (4.6) i	306.5 (6.6) l	232.3 (2.7) m	198.7 (1.3) n	119.4 (2.9) o
	S3	805.3 (1.9) a	727.2 (3.8) b	665.1 (14.9) c	620.2 (4.4) d	543.9 (2.4) e	474.7 (2.2) f	430.3 (15.6) g	393.7 (2.2) h	356.0 (9.7) i	301.6 (7.6) l	253.6 (4.6) m	206.8 (3.9) n	137.3 (2.6) o
	S14	778.9 (3.2) a	670.2 (3.2) b	591.8 (6.7) c	540.4 (6.8) d	512.5 (7.8) e	506.0 (5.2) e	494.7 (5.1) e	475.3 (2.7) f	428.2 (2.8) g	406.2 (2.6) h	330.7 (3.1) i	227.4 (3.1) l	149.8 (4.5) m
	S16	677.8 (1.6) a	592.5 (2.4) b	538.3 (5.9) c	478.5 (11.7) d	465.5 (4.3) d	432.5 (5.0) e	419.2 (5.2) ef	399.6 (5.8) fg	380.2 (4.4) g	345.7 (6.8) h	295.4 (1.5) i	237.8 (2.1) l	164.6 (4.0) m
	S9	616.6 (3.5) a	569.2 (3.3) b	513.3 (2.3) c	408.2 (11.3) d	399.8 (2.3) d	390.5 (4.0) de	372.8 (1.5) ef	354.6 (2.2) fg	341.3 (12.1) g	314.9 (8.8) h	263.9 (1.5) i	218.0 (3.5) l	180.3 (2.7) m
	S15	577.0 (4.2) a	478.1 (7.0) b	380.2 (1.0) c	319.8 (1.5) d	315.6 (3.6) d	312.6 (4.2) de	299.5 (4.1) ef	295.7 (3.6) f	277.7 (2.4) g	266.1 (3.5) g	213.8 (2.3) h	128.4 (4.0) i	81.3 (3.2) l

*The values are expressed as the sum of the following phenols: hydroxytyrosol (3,4-DHPEA), tyrosol (p-HPEA), oleacein (3,4-DHPEA-EDA), oleuropein aglycon (3,4-DHPEA-EA), oleochantal (*p*-HPEA-EDA), ligstroside aglycon (*p*-HPEA-EA) and lignans ((+)-1-acetoxypinoresinol and (+)-pinoresinol). ** The results are the means of two independent determinations ± standard deviation. Different letters in each row indicate statistically different values at $p < 0.05$. Legend: VOOlmp: Virgin olive oil with low-medium poly-phenol content; VOOmhp: Virgin olive oil with medium-high polyphenol content.
